# Investigation of the expression of the EphB4 receptor tyrosine kinase in prostate carcinoma

**DOI:** 10.1186/1471-2407-5-119

**Published:** 2005-09-20

**Authors:** Yen-Ching Lee, Janeanne R Perren, Evelyn L Douglas, Michael P Raynor, Maria A Bartley, Peter G Bardy, Sally-Anne Stephenson

**Affiliations:** 1 Department of Haematology/Oncology, The Queen Elizabeth Hospital, Woodville, South Australia, Australia, 5011; 2 Department of Medicine, University of Adelaide, The Queen Elizabeth Hospital, Woodville, South Australia, Australia, 5011

## Abstract

**Background:**

The EphB4 receptor tyrosine kinase has been reported as increased in tumours originating from several different tissues and its expression in a prostate cancer xenograft model has been reported.

**Methods:**

RT-PCR, western blotting and immunohistochemical techniques were used to examine *EphB4 *expression and protein levels in human prostate cancer cell lines LNCaP, DU145 and PC3. Immunohistochemistry was also used to examine localisation of EphB4 in tissue samples from 15 patients with prostate carcinomas.

**Results:**

All three prostate cancer cell lines expressed the *EphB4 *gene and protein. EphB4 immunoreactivity *in vivo *was significantly greater in human prostate cancers as compared with matched normal prostate epithelium and there appeared to be a trend towards increased expression with higher grade disease.

**Conclusion:**

*EphB4 *is expressed in prostate cancer cell lines with increased expression in human prostate cancers when compared with matched normal tissue. EphB4 may therefore be a useful anti-prostate cancer target.

## Background

Prostate cancer is the most frequent cause of cancer death in men in Australia. Although many genetic changes have been detected in human prostate cancer, the role of most of these in initiation and progression of the disease is unclear. Receptor tyrosine kinases (RTKs) couple ligand binding to downstream signalling cascades and gene transcription and are key regulators of normal cellular processes such as growth, differentiation, migration and apoptosis. RTKs are also critically involved in the development and progression of human cancers and are therefore useful targets for anti-cancer therapies [1,2]. At least 50 RTKs in 20 different families have been identified and the largest family of these contains the Eph receptors [3]. This family in humans currently includes 14 members divided into two classes, designated A and B, based on sequence homology, structure and ligand binding affinity [4,5]. The ligands for the Eph receptors are called ephrins and are anchored on the plasma membrane through either a glycosyl phosphatidylinositol (GPI) link (ephrin-A) or a transmembrane domain (ephrin-B) [6].

Ligand binding induces receptor autophosphorylation of intracellular tyrosine, threonine and serine residues and allows interactions with a variety of different proteins that regulate cell functions such as contact inhibition, cytoskeletal organisation and cell motility [7,8]. Several studies have described intricate signalling networks often important to cell proliferation, migration, survival and differentiation [reviewed in [9]] that centre around Eph receptors and their ligands.

Members of both classes of Eph receptor have been found to have increased gene expression and/or protein levels in tumours from many different human tissues [10-20]. In particular EphB4, located at chromosome 7q22.1, and initially isolated from a human hepatocellular carcinoma cell line Hep3Ba [10] has been reported by us and others to be highly expressed in many tumour tissues including colon [11,12], breast [13,14], endometrium [15,16], lung [17] and head and neck [18,19]. Robinson et al (1996) used degenerate RT-PCR oligonucleotide primers specific to two conserved motifs in the tyrosine kinase domains of 40 different kinases to amplify those kinases that are expressed in a prostate cancer xenograft model [20]. *EphB4 *was identified as one of the RTKs expressed in the xenograft tissue. More recently, Xia et al (2005) report that EphB4 is commonly expressed in prostate tumour tissues and cell lines and knockdown of EphB4 protein using siRNA and antisense approaches inhibited cell growth/viability, migration and invasion both *in vitro *and *in vivo *[21]. Concurrently with Xia et al's study, in this pilot study we also investigated the expression patterns of the *EphB4 *gene and its protein product in prostate cancer cell lines and tumour tissue samples.

## Methods

### Cell culture

Human prostate cancer cell lines LNCaP, DU145 and PC3 were cultured in HEPES-buffered RPMI 1640 medium (Invitrogen, Carlsbad, CA), pH 7.4. The medium was supplemented with 100 U/ml penicillin, 100 μg/ml streptomycin, 160 μg/ml L-glutamine and 10% heat-inactivated foetal bovine serum (JRH Biosciences, Lenexa, KS) in 75 cm^2 ^vented tissue culture flasks at 37°C in a 5% CO_2 _environment. Cells were collected at >90% confluency by trypsin digestion and centrifugation for 5 min at 1000 rpm, resuspended in phosphate buffered saline (PBS) and counted using a haemocytometer.

### RNA extraction from cell lines

RNA was isolated from cell lines using Tri Reagent (Invitrogen). Growth medium was aspirated from the flask of growing cells (>90% confluent) and the cells washed with phosphate-buffered saline (PBS) before being directly lysed using 1 ml of Tri Reagent. After a five min incubation with gentle rocking, the cell lysate was removed to a 2 ml eppendorf tube. The RNA fraction was extracted using the manufacturer's recommendations.

### Protein extraction from cell lines

Growth medium was aspirated from the flask of growing cells (>90% confluent) and the cells washed with phosphate-buffered saline (PBS) before being directly lysed using 1 ml of Cell Lytic M Cell Lysis Reagent (Sigma) supplemented with 5 μl Protease Inhibitor Cocktail (Sigma). Protein lysate was removed to a 1.5 ml microfuge tube and the solution mixed for 30 min at 4°C on a rotary mixer. Insoluble protein was pelleted by centrifugation at 4°C for 30 min and the supernatant containing the soluble proteins was stored at -80°C until required for Western analysis. Protein concentrations were determined using the *DC *Protein Assay Kit from Biorad (Sydney, NSW, Australia) following the manufacturer's protocol and using bovine serum albumin diluted from 0.1 mg/ml to 10.0 mg/ml to determine the standard curve.

### Real-time RT-PCR to determine relative expression of *EphB4*

Total RNA (2 μg) was reverse transcribed at 37°C using 3 μl pD(N)_6 _primers (Invitrogen), 200 μM each deoxyribonucleoside triphosphate (dNTP) (Pharmacia, Uppsala, Sweden) and 200 U Superscript III reverse transcriptase (Invitrogen) in a reaction volume of 30 μl. Primers specific to either *EphB4 *or the housekeeping genes Porphobilinogen deaminase (*PBGD*) and Hypoxanthine phosphoribosyl-transferase 1 (*HPRT 1*) were used in a PCR reaction carried out in a BioRad iCycler MyiQ Real Time thermocycler or a Corbett Rotorgene 3000 (Table I). The following conditions for the PCR reaction were used: 1.5 mM MgCl_2_, 200 μM each dNTP, 50 ng of each primer, and 0.5 units of HotStarTaq DNA polymerase in 1 × PCR buffer (Qiagen, Melbourne, Australia). Cycling conditions included an initial denaturation at 94°C for 15 min, followed by 45 cycles of 94°C for 30 sec, 67°C or 68°C for 30 sec, and 72°C for 30 sec, with a final extension of 72°C for 7 min.

### Western analysis

Proteins (50 μg) from samples extracted from prostate tumour cell lines were separated on duplicate 8% Tris-Glycine iGels (Gradipore, Sydney, Australia). The protein separated on one of the gels was visualised by coomassie staining and the protein on the duplicate gel was electrophoretically transferred to MFS nitrocellulose membrane (Adelab, Adelaide, Australia). Non-specific binding was blocked by incubation for 1 h at room temperature using a Western blocking buffer containing 1% casein in maleic acid buffer (Roche) diluted using TBS with 0.1% Tween-20 (TBS-T). EphB4 antigens were detected using a 1:1000 dilution of a mouse monoclonal antibody raised to human EphB4 (Zymed, CA) in blocking buffer. After a 1 h incubation at room temperature, the primary antibody was detected using a HRP-labelled anti-mouse secondary antibody (Roche) and the ECL Western Blotting System from Amersham Biosciences (Sydney, Australia) following the manufacturer's recommendation. Blots were exposed to Hyperfilm™ECL™ (Amersham Biosciences) for between 5 and 30 sec. The chemiluminescence was removed from the blot by incubation in a stripping solution containing 100 mM β-mercaptoethanol, 2% SDS and 62.5 mM Tris-HCl pH 6.7 for 20 min at 50°C with gentle agitation. The filter was then washed with TBS-Tween 20 before blocking and re-probing with a mouse anti-α-actin monoclonal antibody (Chemicon, Temecula, CA) then it was stripped again and probed with a rabbit anti-calnexin antibody (Sigma) for loading comparison.

### Immunofluorescence of cells grown on slides

Cell were grown to >50% confluence in the wells of 8 well chambered slides. Before staining, the medium was aspirated and the cells washed gently with PBS. Cells were then fixed in 4% paraformaldehyde and washed well before non-specific binding sites were blocked with 3% serum in PBS for 20 min at room temperature. Cells were then incubated overnight at 4°C with a 1:200 dilution of the EphB4-specific rabbit polyclonal antibody H-200 (Santa Cruz Biotechnology, Santa Cruz, CA). After rinsing with PBS, the sections were incubated with an Alexa Fluor 488 goat anti-rabbit IgG (H+L) (Molecular Probes). Immunofluorescence was visualised using a TE2000E microscope with a C1 confocal scanning head (Nikon, Tokyo, Japan).

### Tissue immunohistochemistry

Four consecutive 8 μm sections of formalin-fixed paraffin-embedded tissue from 15 different patients with prostate cancer were a gift from Dr Michael Brown, Medical Oncology, Royal Adelaide Hospital, Adelaide, Australia. A fifth slide, stained to visualize the histological features of the tissue, was used to identify the foci of tumour tissue within the adjacent normal prostate tissue by a trained pathologist. The paraffin was removed by incubation in Histoclear (National Diagnostics, Atlanta, Georgia) and the section re-hydrated in ethanol before antigen retrieval by boiling in 10 mM citric acid pH 6 for 10 min. Sections were cooled, then washed in PBS before removal of endogenous peroxidase activity by incubation in 0.5% hydrogen peroxide/methanol for 30 min at room temperature. Non-specific binding sites were blocked with 3% normal goat serum in PBS for 20 min at room temperature and the Vector Laboratories (Burlingame, CA) Avidin/Biotin blocking kit following the manufacturer's instructions. The sections were then incubated overnight at 4°C with a 1:200 dilution of the H-200 EphB4-specific antibody (Santa Cruz Biotechnology). After rinsing with PBS, the sections were incubated with biotinylated goat anti-rabbit IgG (Vector Laboratories) for 30 min at room temperature followed by washing with PBS. Immunoreactivity was detected with the avidin-biotin system (Vector Laboratories) using 18.5 mM 3,3'-diaminobenzidine tetrahydrochloride (Sigma) as a chromogen for 2 min. The sections were then counterstained using Harris haematoxylin, dehydrated, cleared using SUB-X clearing solution (Surgipath Medical Industries, Inc. Richmond, IL) and mounted using Entellan New (Merck, Darmstadt, Germany). Sections were visualised and images recorded using a Nikon Eclipse E800 microscope with a Spot Camera 2.3.1 (Diagnostic Instruments) and Spot Advanced Version 3.5 software.

## Results

### Relative expression of *EphB4 *in prostate cancer cell lines

**Figure 1:**
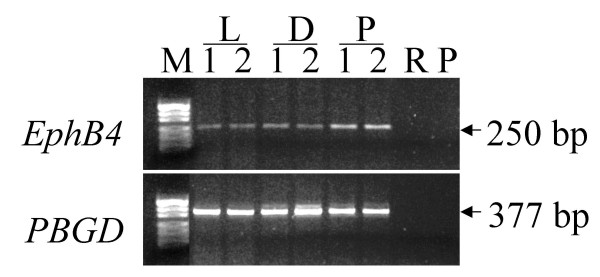
**RT-PCR analysis of *EphB4 *expression in prostate cancer cell lines**. RT-PCR analysis of *EphB4 *and *PBGD *expression in duplicate RNA samples from the three prostate cancer cell lines LNCaP, DU145 and PC3. Sizes of the amplified products are shown on the right (bp). An RNA sample to which no reverse transcriptase was added and a PCR reagent only (containing no template) amplification were also performed as negative controls for each stage of the RT-PCR.

**Figure 2:**
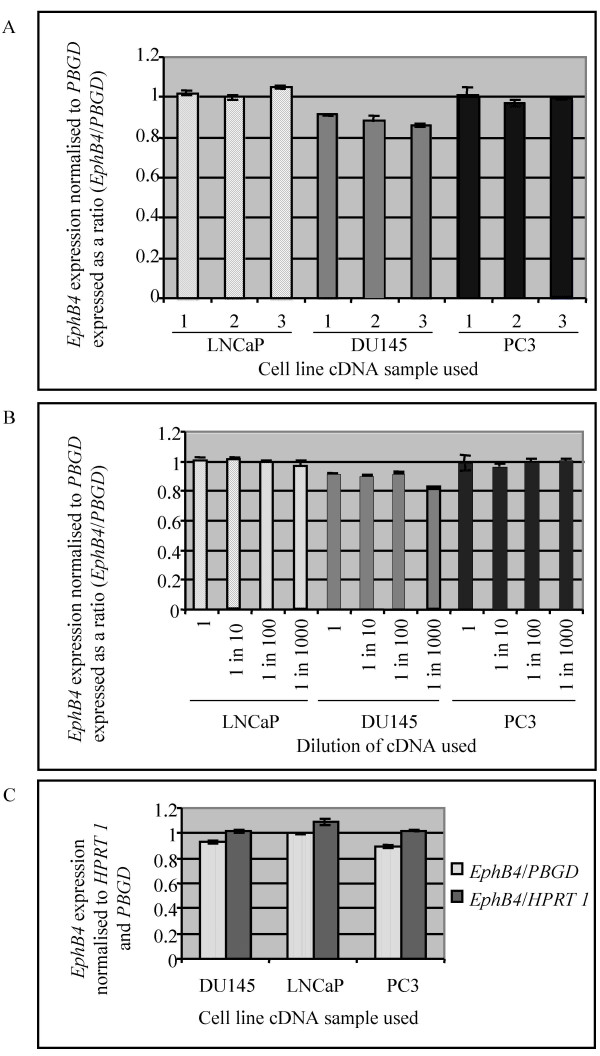
**Normalisation of *EphB4 *expression to housekeeping genes *PBGD *and *HPRT1***. *EphB4 *expression normalised to *PBGD *in prostate cancer cell lines LNCaP, DU145 and PC3. (A) Triplicate amplifications of *EphB4 *and *PBGD *in three samples for each cell line were analysed to determine the relative levels of *EphB4 *expression using the BioRad iCycler. All *EphB4*/*PBGD *ratios were close to 1 indicating that there are comparable amounts of both templates in each RNA samples. (B) Dilutions of a single RNA sample for each cell line were also amplified in triplicate with both gene primer sets to confirm that the ratio was consistent regardless of starting template concentration. (C) Triplicate amplifications of *EphB4*, *PBGD *and *HPRT1 *in three samples for each cell line using the Corbett Rotorgene confirmed the previous result for *EphB4*/*PBGD *and showed a similar result when *EphB4 *expression was normalised to a second housekeeping gene.

The relative expression of *EphB4 *in three prostate cancer cell lines was determined using reverse transcription and real time PCR using two different real-time PCR machines. Cell lines used included the androgen-dependent line LNCaP derived from a lymph node biopsy (moderately differentiated), the androgen-independent line DU145 from a central nervous system metastasis (moderately differentiated with foci of poorly differentiated cells) and the androgen-independent line PC3 from a bone metastasis (poorly differentiated) [22-24]. For each experiment performed amplification reactions were resolved on agarose gels to confirm products of the predicted size for each gene were amplified (example shown in Figure 1). In the first instance, the *EphB4 *gene and the house-keeping control gene *PBGD *were amplified in triplicate from cDNA made using three different RNA extractions for each cell line (Figure 2A) and a serial dilution of one of these cDNA preparations (Figure 2B) using a BioRad iCycler MyiQ. In the second experiment, a second housekeeping gene, *HPRT 1*, was amplified in addition to *EphB4 *and *PBGD *using a Corbett Rotorgene 3000 (Figure 2C). For each individual sample, *EphB4 *expression level was normalised to the expression of *PBGD *and *HPRT 1 *in these samples (*EphB4*/*PBGD *or *EphB4/HPRT 1*). Comparison of the combined data for each cell line showed that there was no statistical difference in the level of *EphB4 *expression in the three prostate cancer cell lines (Figure 2). In a third experiment, RNA was extracted from 6 replicates of cells grown to either full confluence or 50–80% confluence and *EphB4 *expression again normalised to both *PBGD *and *HPRT 1. *There was no statistically significant difference in the level of expression of *EphB4 *in the non-confluent and confluent cells for any of the three cell lines (data not shown).

In these experiments the ratios of *EphB4*/*PBGD *and *EphB4/HPRT 1 *were all close to 1 indicating that there were comparable amounts of *EphB4 *and *PBGD*/*HPRT 1 *templates in each cell line RNA sample. There was no statistical difference between the ratios obtained using serial dilutions of a single cDNA sample for each cell line showing that the amplification is consistent regardless of the starting amount of template. These results support the use of both *PBGD *and *HPRT 1 *as normalisation controls for the *EphB4 *message.

### Western analysis of EphB4 protein in prostate cancer cell lines

**Figure 3:**
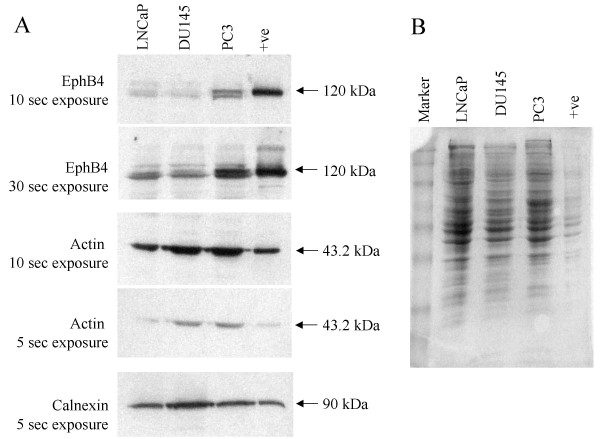
**Western analysis of EphB4 in prostate cancer cell lines**. Western analysis of 20 μg EphB4 protein in prostate cancer cell lines LNCaP (L), DU145 (D) and PC3 (P). (A) The immunoblot was incubated sequentially with antibodies specific to EphB4, α-actin and calnexin and exposed to autoradiographic film for the indicated times. MCF10A engineered to express EphB4 (5 μg protein lysate) was used as a positive control. The arrow indicates the expected size of each protein – 120 kDa for EphB4, 43.2 kDa for α-actin and 90 kDa for calnexin. The size of the marker proteins is shown on the left in kDa. (B) Coomassie stained duplicate gel for loading comparison.

Protein was isolated from the three prostate cancer cell lines for western analysis to determine whether the level of gene expression correlated with the amount of protein present. Duplicate samples were run on two separate gels, one of these was immunoblotted and the other stained with coomassie blue to confirm protein integrity and consistency in the amount of protein loaded. The predicted size of the mature EphB4 protein is 120 kDa and a band of this approximate size was clearly visible in each sample after immunoblotting with an EphB4-specific antibody (Figure 3A). The non-transformed breast cell line MCF10A engineered to over-express *EphB4 *under control of the constitutive CMV promoter was used as a positive control (labelled +ve in Figure 3). In the prostate cell lines a second band of a slightly smaller size is also clearly visible and this may represent an alternatively modified (phosphorylated/glycosylated) or spliced form of EphB4. The coomassie staining of the duplicate gel shows that similar amounts of protein from each cell line was loaded and suggests that the PC3 cell line produces more EphB4 protein than LNCaP than DU145 (Figure 3B). To confirm efficient transfer the blot was also incubated sequentially with an antibody recognizing α-actin. Although a 10 sec exposure time suggested there were similar levels of protein present, a shorter exposure showed differences in the relative amount of actin in each protein lysate. Therefore a second control antibody recognising the ER chaperone calnexin was also used. There was little difference in the relative amounts of calnexin in each sample.

### Immunological analysis of expression of EphB4 in prostate cancer cell lines

**Figure 4:**
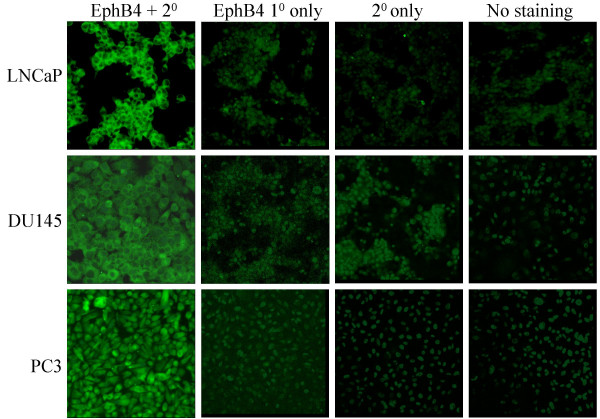
**Immunofluorescent staining of EphB4 in prostate cancer cell lines**. Immunofluorescent staining of EphB4 in prostate cancer cell lines showing diffuse staining on the surface and in the cytoplasm of LNCaP and DU145 and in the cytoplasm only of PC3. There was no reactivity to the primary antibody (EphB4 1° only) or secondary antibody (2° only) alone and little background fluorescence (No staining).

The expression and localisation of EphB4 in the three cell lines grown on slides was determined using immunofluorescence with an EphB4-specific polyclonal antibody. In LNCaP and DU145 cells EphB4 appeared to be present on both the cell surface and in the cytoplasm but in PC3 cells the EphB4 appeared to be mainly cytoplasmic (Figure 4). This cytoplasmic localisation of EphB4 in tumour cells contrasts with its known localisation within sites of cell-cell contact between non-transformed epithelial cells and may suggest that active EphB4 signalling has caused endocytosis of the receptor-ligand complex in these cells. Similar results have been reported by Wu et al (2004) who also used the EphB4 antibody sc-5536 from Santa Cruz Biotechnology in their study of EphB4 expression in breast cancer cell lines and tissue samples [14]. All four breast cancer cell lines examined showed strong immunostaining in the cytoplasm.

### Immunohistochemical analysis of expression of EphB4 in primary prostate tumours

**Figure 5:**
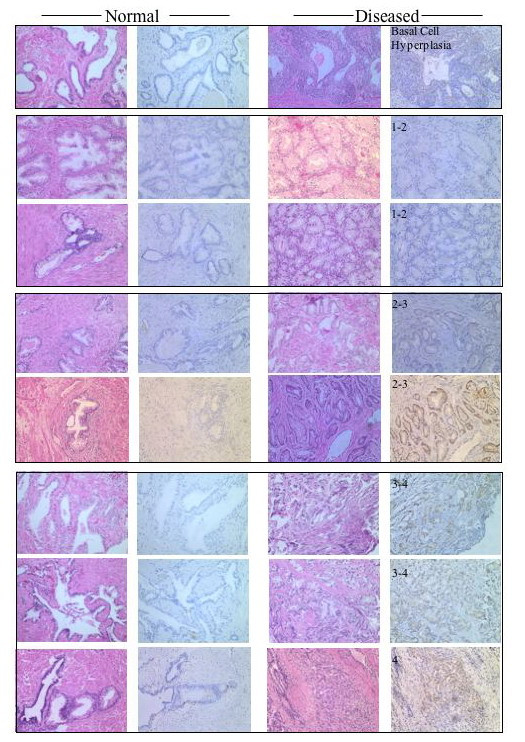
**Immunohistochemical staining of EphB4 in prostate cancer samples**. The expression of EphB4 in human prostate carcinomas was detected using immunohistochemical staining of formalin fixed, paraffin-embedded tissue samples. Images (20× magnification) of normal (panel 2) and diseased tissue (panel 4) from eight different patient samples are shown with a Haematoxylin/eosin stained consecutive section (panels 1 and 3). The Gleason score for each disease focus is shown in the top left-hand corner of the image of each tumour focus. A single sample that contained a focus of basal cell hyperplasia, two samples with well-differentiated adenocarcinoma, two samples of moderately differentiated adenocarcinoma and three samples of poorly differentiated adenocarcinoma are shown. The brown stain from the biotinylated secondary antibody indicates the EphB4 protein. Nuclei are stained with Harris haematoxylin and appear blue. Staining of normal tissues was either absent or weak and diffuse. Increased staining of the tumour tissue appeared to correlate with increased Gleason score. There was no cross-reactivity with the secondary antibody alone (result not shown).

To localize the expression of EphB4 in normal prostate and tumour tissue, we performed immunohistochemistry using an EphB4-specific antibody on 15 prostate tumour samples collected by transurethral resection. These patient samples were chosen because they contained foci of prostate cancer or benign prostatic hyperplasia within the normal tissue. The results for 8 representative patient sample sets are presented in Figure 5. The epithelial cells lining the ducts in the normal tissue can be clearly distinguished by the rows of regular nuclei stained blue with Harris Haematoxylin. There was either no or only very weak diffuse staining of the normal epithelial cells, stroma and endothelial cells of blood vessels indicating little immunoreactivity to the EphB4 antibody. There was no staining with an isotype matched IgG antibody or the secondary antibody alone in either normal or tumour tissues (results not shown).

**Figure 6:**
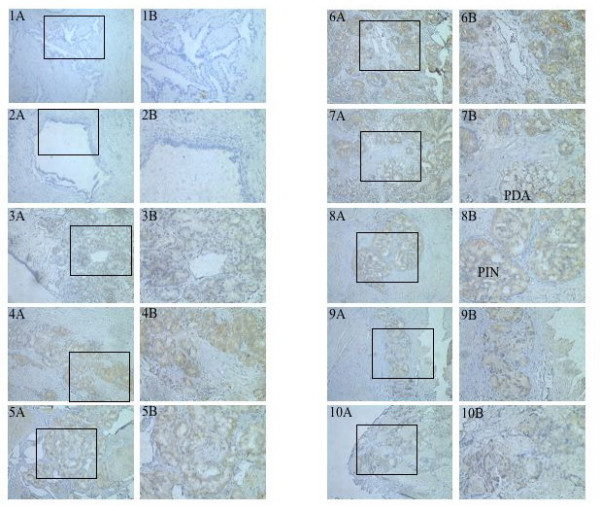
**Immunohistochemical staining of EphB4 in several foci of a single prostate cancer sample**. The expression of EphB4 a different foci in a single patient sample was compared by immunohistochemistry. Images from 10× magnification (A) and a region from this (as indicated by the box) at 20× magnification (B) are shown for 2 regions of normal prostate duct (1 and 2) and 8 regions with disease (3–10) from this single sample. A comparable level of EphB4 staining was seen in regions of high grade PIN (eg. focus 8 indicated PIN) and regions of poorly differentiated adenocarcinoma (eg. focus 7 indicated PDA). Regions of well-differentiated (eg focus 3) and moderately differentiated (eg focus 10) also show comparable staining.

Foci of prostate cancer within these samples showed brown staining indicating immunoreactivity to the EphB4 antibody and therefore the presence of the EphB4 protein. A single patient sample had a low level of staining associated with foci of basal cell hyperplasia. In this case EphB4 may be involved in a proliferative role [25]. There also appeared to be an increase in the amount of staining associated with an increase in Gleason score. There was little staining in the 5 different patient samples that contained foci of well-differentiated adenocarcinoma (2 examples are shown in Figure 5), and increased staining in the three patients with foci of moderately differentiated and the three patients with poorly differentiated adenocarcinomas. Because prostate cancer is multifocal, each sample was examined to determine if it contained foci of different grades and whether immunoreactivity differed in intensity within these. As these samples were collected during transurethral resection to debulk enlarged prostates, and were later found to contain foci of disease, only a few samples contained foci of different grades. One patient in particular had large regions classified clinically as prostatic intraepithelial neoplasia (PIN) and staining within these foci was surprisingly of equal intensity to the regions of poorly differentiated adenocarcinoma (Figure 6).

## Discussion

In recent years, experiments using monoclonal antibodies with limited normal-tissue reactivity have indicated that these are good candidates for development as therapeutic agents against cancer. *EphB4 *is a receptor protein tyrosine kinase (RTK) that is dramatically up-regulated on many epithelial cancers, displays limited expression on normal adult tissue and would therefore appear to be a potential target for monoclonal therapies. EphB4 was identified by Robinson et al (1996) as one of several RTKs that were expressed in a xenograft model of prostate cancer [20] and until recently this was the only report of any link between EphB4 expression and prostate cancer.

During the review of this article, Xia et al (2005) presented results showing that EphB4 is increased in 66% (41/62) of prostate tumours tested with low intensity expression in only 15% (3/20) normal prostate samples [21]. Our western analysis of PC3, LNCaP and DU145 confirmed Xia et al's results [21], with higher amounts of protein present in PC3 lysates that LNCaP and DU145. In the case of DU145 this can perhaps be explained by regulation of *EphB4 *gene expression as real-time PCR using normalisation to two different house-keeping genes demonstrated that there was approximately 20% less *EphB4 *transcript expressed in the DU145 cell line than in LNCaP and PC3 (P < 0.05). However, as there was no significant difference in the activity of the gene in LNCaP and PC3, it is possible that EphB4 may also be regulated post-transcriptionally. The presence of a second band detected by western analysis with the EphB4-specific antibody may also indicate that EphB4 is regulated post-translationally and this needs to be explored in more detail.

Although Xia et al's study of cell lines also indicated an association between the level of EphB4 expression and aggressive growth, they did not report a direct correlation between increased EphB4 expression with higher grade of the tumour tissue samples. We examined a panel of 15 prostate cancer clinical specimens collected by transurethral resection. The prostate tumour samples we examined contained both normal prostate and tumour foci and this enabled a comparison of EphB4 immunoreactivity in normal prostate and tumour cells simultaneously from the same patient and the determination of whether the level of EphB4 also correlates with histological grade and/or stage of prostate carcinoma. Using immunohistochemical techniques, we showed that EphB4 is produced in increased amounts in human prostate cancer tissue compared with adjacent normal tissue and that this immunoreactivity was associated with the tumour epithelial cells themselves. There also appeared to be trend towards an increasing level of EphB4 protein in the tumours from the well-differentiated to the moderately and poorly differentiated cancers.

A positive correlation between histological grade, stage of carcinoma and level of EphB4 protein has also been reported in breast and endometrial carcinoma [13-16]. EphB4 has been reported to be elevated in breast primary infiltrating ductal carcinomas with a high grade of malignancy [13] and recently Wu et al (2004) reported that EphB4 protein expression in 94 tumour samples was positively associated with increased clinical stage and histological grade [14]. Sakano et al, (1996) suggested that the balance of EphB4 and its preferred ligand ephrin-B2 is disrupted when mammary epithelial cells become transformed [25] and loss of ephrin-B2 expression has been associated with an increase in *EphB4 *expression [26]. However contrary to this Berclaz et al (2002) report a highly significant correlation between EphB4 positivity and low histological grade [27]. One of the patients examined here also showed a comparable level of EphB4 staining in foci of PIN and poorly differentiated adenocarcinoma. If the PIN is a precursor to adenocarcinoma in this case, this might suggest that increased expression of EphB4 was an early event in the development of this patient's disease. Furthermore, a single focus of basal cell hyperplasia in a second patient did show a low level of EphB4 immunoreactivity and this may indicate a role for EphB4 in proliferation of these cells [25].

The results presented here suggest that elevated levels of *EphB4 *are relevant to prostate cancer but despite substantial evidence in the literature that EphB4 has an important role in progression of many epithelial tumours, the mechanism by which these receptors contribute to tumorigenesis is still being resolved. In only a very few cases have Eph receptors been demonstrated to have transforming potential [28-30]. In particular, a study reported by Zelinski et al (2002) showed that *EphA2 *expression transforms MCF10A cells as judged by conversion to a fibroblastic morphology, growth in soft agar and the ability to engraft and metastasise in a nude mouse model [29].

Although similar experiments over-expressing EphB4 in non-transformed cell lines have yet to be reported, a study by Munarini et al (2000) using transgenic mice expressing *EphB4 *and *neuT *suggested that *EphB4 *over-expression by itself is not tumorigenic but provides convincing evidence that it favours an invasive/metastatic phenotype [31]. Although mammary tumours were not observed in the EphB4 transgenic animals, in double transgenic animals expressing both *EphB4 *and *neuT*, tumour appearance was significantly accelerated relative to *neuT*-only animals, and in addition metastases were observed in the lung. It was not clear if this was an effect of EphB4 on metastasis or a result of accelerated tumour growth but these results clearly implicate over-expression of *EphB4 *in tumour growth and/or establishment of the invasive phenotype in the adult mammary tumours. While the single transgenic *EphB4 *animals did not develop tumours during this experiment it is possible that these studies were not taken out far enough to conclude that *EphB4 *over-expression is insufficient to induce transformation with a long latency.

Xia et al's (2005) recent experiments targeting EphB4 using siRNA to knockdown *EphB4 *expression *in vitro *have shown that EphB4 is involved in growth/viability, migration and invasion of prostate cancer cell lines and supports Munarini et al's study [21]. Furthermore, EphB4 antisense oligonucleotides were given intraperitoneally to nude mice bearing PC3 xenografts in the posterior prostate (n = 6), tumours were fewer and smaller, with increased apoptosis and reduced microvascular density than sense- or diluent-treated control animals. The reduction in microvascular density is particularly interesting given that in other epithelial tumours, EphB4-positive cells are often found in regions that are rich in capillaries [13] and it has been suggested that EphB4 over-expression in tumours may promote tumour growth by facilitating angiogenesis.

A direct role for EphB4 in tumour angiogenesis has been suggested by experiments showing that A375 melanomas form smaller tumours in the presence of soluble EphB4 [32] possibly because the soluble EphB4 interferes with binding of endogenous EphB4 on tumour cells to ephrin-B2 on endothelial cells. A role in angiogenesis would be consistent with the normal role for EphB4 in vascular development and remodelling demonstrated in mouse and *Xenopus *[33,34]. Noren et al (2004) contradict this finding in a study that found that increased expression of a signaling-defective form of EphB4 in breast cancer cells (dominant negative) was still able to make tumour xenografts grow more rapidly [35]. This may be because the EphB4 ectodomain exerted a chemoattractive effect on endothelial cells through ephrin-B2 expressed on these cells and promoted endothelial cell proliferation and survival resulting in more tumour vasculature. However, Noren et al applied a high concentration of soluble ephrin-B2 Fc which is promiscuous and signals other Eph receptors (particularly EphB2) but the phosphorylation of other EphRs was not determined in this study.

## Conclusion

Further investigation is needed to determine the roles of EphB4 in prostate cancer development and progression. The trend of increasing EphB4 reactivity toward higher grade disease seen in this pilot study might suggest that it is more important in the later stages of the tumour development such as metastasis and further investigation of EphB4 in the development of prostate cancer is warranted. Therapies that target EphB4 may prove to be successful in preventing the metastatic spread of the disease.

## Competing interests

The author(s) declare that they have no competing interests.

## Authors' contributions

YCL carried out most of the studies with the assistance of JP and ED and drafted the manuscript. JP and ED also participated in the analysis and interpretation of the data and revision of the manuscript. MR optimised the real-time PCR experiments, participated in the analysis and interpretation of the data and revision of the manuscript. MB optimised the Western analysis, participated in the analysis and interpretation of the data and revision of the manuscript. PB participated in the analysis and interpretation of the data and revision of the manuscript. SS conceived of and co-ordinated the study and helped to draft the manuscript. All authors read and approved the final manuscript.

## Pre-publication history

The pre-publication history for this paper can be accessed here:



## Figures and Tables

**Table 1:** Primer sequences used to amplify *EphB4 *and the control housekeeping genes *PBGD *and *HPRT 1 *with expected size of the corresponding PCR product in base pairs (bp) and the annealing temperature used in the reaction.

Primer	Sequence 5'-3'									Product	Annealing
EphB4 F	CCC	CAG	GGA	AGA	AGG	AGA	GCT	G			
EphB4 R	GCC	CAC	GAG	CTG	GAT	GAC	TGT	G		250 bp	68°C
PBGD F	CTT	TCC	AAG	CGG	AGC	CAT	GTC	TGG			
PBGD R	CAT	GAG	GGT	TTT	CCC	GCT	TGC	AGA		377 bp	68°C
HPRT F	GGC	TAT	AAA	TTC	TTT	GCT	GAC	CTG	CTG		
HPRT R	CAA	AGT	CTG	CAT	TGT	TTT	GCC	AGT	GTC	230 bp	67°C
